# Elevated Serum Cystatin C and Decreased Cathepsin S/Cystatin C Ratio Are Associated with Severe Peripheral Arterial Disease and Polyvascular Involvement

**DOI:** 10.3390/diagnostics12040833

**Published:** 2022-03-28

**Authors:** Előd Ernő Nagy, Attila Puskás, Piroska Kelemen, Katalin Makó, Zoltán Brassai, Jolán Hársfalvi, Attila Frigy

**Affiliations:** 1 Department of Biochemistry and Environmental Chemistry, George Emil Palade University of Medicine, Pharmacy, Sciences and Technology of Targu Mures, 540142 Targu Mures, Romania; 2 Laboratory of Medical Analysis, Clinical County Hospital Mures, 540394 Targu Mures, Romania; 3 Angio-Center Vascular Medicine, 540074 Targu Mures, Romania; puskasat@gmail.com; 4 Department of Internal Medicine II, George Emil Palade University of Medicine, Pharmacy, Sciences and Technology of Targu Mures, 540142 Targu Mures, Romania; piroska_kelemen@yahoo.com (P.K.); makokatalin@yahoo.com (K.M.); abrassai@yahoo.com (Z.B.); 5 II Clinic of Internal Medicine, Emergency Clinical County Hospital Targu Mures, 540142 Targu Mures, Romania; 6 Hestia General Practioner Ltd., H-1188 Budapest, Hungary; 7 Department of Biophysics and Radiation Biology, Faculty of Medicine, Semmelweis University, H-1444 Budapest, Hungary; harsfalvi.jolan@med.semmelweis-univ.hu; 8 Department of Internal Medicine IV, George Emil Palade University of Medicine, Pharmacy, Sciences and Technology of Targu Mures, 540142 Targu Mures, Romania; afrigy68@gmail.com; 9 Department of Cardiology, Clinical County Hospital Mures, 540072 Targu Mures, Romania

**Keywords:** cystatin C, cathepsin S, atherosclerosis, peripheral arterial disease, ankle–brachial index, polyvascular disease

## Abstract

Peripheral arterial disease (PAD) is frequently associated with atherosclerotic manifestations of the carotids and coronaries. Polyvascular involvement and low ankle–brachial index predict major cardiovascular events and high mortality. Cathepsin S (Cat S) promotes the inflammatory pathways of the arterial wall, while Cystatin C (Cys C) functions as its inhibitor; therefore, Cys C was proposed to be a biomarker of progression in PAD. In a single-center observational study, we investigated the correlations of serum Cys C and Cat S/Cys C ratio in a group of 90 PAD patients, predominantly with polyvascular involvement. Cys C and Cat S/Cys C were associated with ankle–brachial index (ABI) scores <0.4 in univariate and multiple regression models. Furthermore, both markers correlated positively with the plasma Von Willebrand Factor Antigen (VWF: Ag) and Von Willebrand Factor collagen-binding activity (VWF: CB). In addition, Cat S/Cys C was significantly decreased, whereas Cys C increased in subjects with three-bed atherosclerotic involvement. According to our results, high serum Cys C and low Cat S/Cys C ratios may indicate severe peripheral arterial disease and polyvascular atherosclerotic involvement.

## 1. Introduction

Structural remodeling of the arterial wall is a key-importance pathway of atherosclerosis. From a macroscopic point of view, there are two distinct ways of this phenomenon: the constrictive type narrows the arterial lumen and is determined by intimal hyperplasia and the ingrowing mass of the atherosclerotic plaque, whereas for the expansive type, de novo remodeling is related to plaque vulnerability [1]. Vascular proteolytic enzymes have an essential role in biological processes common to atherosclerosis and its complications: thrombosis and aneurysm formation. These enzymes are studious actors in vessel wall rearrangement; they influence elasticity, endothelial phenotype, and barrier function by degrading essential elements of the extracellular matrix: elastin, collagen, and fibronectin [2].

Cathepsins are potent lysosomal proteases degrading various target proteins and peptides, especially extracellular matrix elements. In the cathepsin enzyme family, 11 cysteinyl proteases were classified: cathepsin B, C, F, H, K, L, O, S, V, W, and Z. Cathepsin S is known for its intense elastolytic activity and plays a pivotal role in the remodeling of the arterial wall in the course of atherosclerosis [3]. The enzymatic and non-enzymatic modifications of low-density lipoproteins (LDL) and the accumulation of desialylated and oxidized LDL formed in foam cells trigger a strong intracellular response at the NLRP3 inflammasomes, shifting macrophages to a pro-inflammatory M1 phenotype [4,5]. In stable plaques, the M1-type, whereas in unstable plaques with intense remodeling, M2-type macrophages seem to be the primary source of cathepsins [6].

In contrast with the normal vessel wall, increased cathepsin S, K, and L expression have been found in advanced atherosclerotic lesions [7]. Smooth muscles cells and infiltrating macrophages secrete cathepsins in response to pro-inflammatory stimuli, such as IL-1β or IFNγ, and these have a crucial role in the degradation and reparation of the extracellular matrix. Cathepsin S deficiency on LDL receptor −/− or apoE −/− background manifests reduction of atherosclerotic plaque size and development [7].

Cystatin C is a low molecular weight, active cysteine protease inhibitor of 13.34 kDa, almost ubiquitously produced by many cells, abundant in all body fluids [8]. The molecule has been proposed to be a strong predictor of incident or recurrent cardiovascular events. Early studies found reduced Cys C expression in atherosclerotic and aneurysmal arterial walls, in contrast with healthy blood vessels [9]. Decreased quantities of circulating Cys C were associated with coronary artery ectasia and abdominal aortic aneurysm. In contrast, the elevation of serum Cys C, especially on the background of systemic hypertension, is significantly associated with fatal and non-fatal cardiovascular events [10]. Experimental studies confirmed the reduction of Cys C content in atherosclerotic and aneurysmal arterial specimens. Apo E −/− Cys C −/− mice show increased SMC accumulation, fragmentation of tunica media, greater plaque size, and macrophage content [11].

Conversely, in the first week after cerebral infarction, the cathepsin S and the cathepsin S/cystatin C ratios are significantly elevated [12]. In the Cardiovascular Health Study with an 8-year follow-up, cystatin C proved to be a significant predictor of all-cause and cardiovascular death (myocardial infarction and stroke). Arpegard et al. revealed that cystatin C is an independent marker of peripheral arterial disease after adjustment with estimated glomerular filtration rate (GFR) and correlates with inflammation, IL-6, and CRP [13]. High cystatin C was independently associated with future PAD events and outcomes in community-dwelling elderly. History of coronary disease, stroke, and hypertension showed a significant increasing trend with the cystatin C quintiles [14].

Peripheral arterial disease (PAD) develops highly on a generalized atherosclerotic background. Atherosclerotic manifestations in more than two arterial beds (lower limb arteries, carotids, coronary arteries) define polyvascular disease. This state confers an increased risk of cardiovascular mortality, stroke, and myocardial infarction in observational studies (CRUSADE, REACH) and randomized clinical trials (CAPRIE, PEGASUS-TIMI 54, EUCLID). These studies also revealed a gradual increase in major cardiovascular events [15]. In some studies, polyvascular atherosclerotic manifestations, as coexisting PAD and CAD (coronary artery disease), showed significantly higher cystatin C values than PAD or CAD alone. Fung et al. set a biomarker panel to predict hemodynamically significant PAD + CAD versus hemodynamically non-significant PAD or CAD alone and found cystatin C along with β2-microglobulin, hsCRP, and fasting glucose to be strong predictors [16].

In many centers engaged in primary vascular care, PAD diagnosis, besides personal history and objective examination, relies on the ankle–brachial pressure index (ABI) determination, a widely used, non-invasive, and reproducible approach. ABI can be considered a measure of atherosclerotic burden; however, some authors suggest it does not permanently mark the severity of functional impairment in PAD as of the pain-free walking distance [17].

Other studies demonstrated lower ABI values with more severe functional impairment and faster functional decline. Further, ABI is an independent indicator of atherothrombotic events occurring outside of the limbs, related to all-cause mortality in men and women [18]. The correlation of ABI histogram versus all-cause and cardiovascular mortality is U-shaped, with maximum mortality rates at the lowest and highest ABI values [19]. In the current study, we proposed to analyze the serum Cat S and Cys C levels and the Cat S/Cys C ratio in a cohort of patients with polyvascular atherosclerotic disease, hospitalized primarily for PAD symptoms. We classified our cases according to their ABI and the number of arterial beds affected by atherosclerosis, characterizing the between-group differences, the in-group correlations, and analyzing the importance of the biomarkers in determining low ABI values and severe polyvascular disease with three-bed involvement.

## 2. Materials and Methods

### 2.1. The Study Cohort

For the study, 103 patients diagnosed with various stages of PAD were selected. After excluding patients with malignancies, acute inflammatory disorders, infections, or autoimmune vascular disease, 90 subjects—68 males and 22 females (median age 66 years)—were enrolled. An individual written consignment was obtained in every patient, and the study was approved by the ethics committee of the hospital (15/200307). Each patient underwent systematic data collection concerning their routine demographic, clinical, laboratory, and echocardiographic data. Hypertension was diagnosed according to JNC 7 protocols [20]. Diabetes mellitus was defined as fasting glucose ≥7 mmol/L on at least two measurements or current use of insulin or oral antidiabetic medication.

The estimated glomerular filtration rate was calculated using the Modification of Diet in Renal Disease (MDRD) formula [21].

### 2.2. ABI and Fontaine Staging

Diagnosis of PAD was stated according to the patients’ medical history, clinical examination, and measurement of the ABI and Duplex Doppler ultrasonography. For calculation of the ABI, in the supine position of the patient, and after 10 min of rest, bilateral systolic blood pressure measurements were performed on the brachial, dorsalis pedis, and posterior tibial arteries, using a standard sphygmomanometer (with an aneroid manometer) and a 7 MHz continuous wave (CW) Doppler device. For each limb, the higher systolic pressure (out of the values obtained at the dorsalis pedis and the posterior tibial artery) was divided by the higher value of the two brachial systolic pressures. The lower ABI value was used for statistical calculations.

Intermittent claudication was appreciated by completing the Walking Impairment Questionnaire. Stages III and IV, the presence of rest pain and/or ulceration and/or gangrene were considered as critical ischemia of the leg.

### 2.3. Diagnosis of CAD

Coronary artery disease and previous myocardial infarction (MI) were diagnosed based on positive coronary angiography with or without percutaneous angioplasty.

### 2.4. Carotid Ultrasound

Duplex Doppler carotid artery scan was performed using a GE Agilent Image Point HXB.1 Sonos 4500/5500B.1 ultrasound system or a Philips Epiq7 ultrasound machine (Philips Ultrasound, Inc., Bothell, WA, USA). B-mode, color- and pulsed-wave Doppler analyses were performed on both sides to identify arterial wall lesions, stenoses, and occlusions. Stenosis was expressed as the percentage decrease in artery diameter, using the ECST criteria [22].

Where no plaques were identified, carotid intima-media thickness (IMT) was measured bilaterally at the far wall of the common carotid artery, immediately proximal to bifurcation, in end-diastole. The higher IMT value was introduced into the statistic calculations. Carotid artery atherosclerosis was considered to be present when an IMT > 1.00 mm, or the presence of plaques at any level was found.

### 2.5. Biochemical Analysis

Blood samples were collected after overnight fasting into vacutainer tubes with no additive or 3.2% trisodium citrate. The tubes were centrifuged at 3000 rpm for 10 min, after which the serum and plasma aliquots were separated and submitted to routine analysis or deep-frozen at −70 °C. Total cholesterol, HDL cholesterol, and serum triglycerides were measured on an ABBOTT AEROSET biochemistry analyzer. We calculated LDL cholesterol by the Friedewald formula. HsCRP was determined by latex-enhanced turbidimetry on Konelab 30i (Thermo Scientific, Vantaa, Finland). Plasma fibrinogen was measured through a Clauss method with Fibrinogen Reagent (Technoclone, Vienna, Austria) on Behnk Thrombotimer (Norderstedt, Germany). VWF: Ag was measured by a sandwich ELISA according to a previously specified protocol [23], VWF: CB was measured as Ag, applying type III collagen (MilliporeSigma, Milwaukee, WI, USA) to coat the high binding capacity plate (Greiner Bio-One, Kremsmünster, Austria). Serum CysC was analyzed by immunoturbidimetry on Konelab 30i, whereas serum Cat S was determined by a sandwich ELISA assay (ab155427, Abcam, Cambridge, UK), in conformity with the manufacturer’s recommendations.

### 2.6. Statistical Analysis

Data distribution characteristics were analyzed by the Lilliefors and Shapiro–Wilk tests. For between-group comparisons, we applied nonparametric statistical tests: Mann–Whitney U test, and Spearman rank correlation analysis. Categorical variables were analyzed for absolute and relative frequency and were compared with Fisher’s exact test. We performed univariate regression analysis for the individual parameters and set up non-linear logistic regression models for the prediction of the low ABI values and the presence of polyvascular disease. Data processing was performed using Microsoft Excel 2016 and GraphPad Prism 9.03.

## 3. Results

### 3.1. Study Group Characteristics

In total, 68 male and 22 female patients were enrolled in the study group, with a median age of 66 years. Half of the patients suffered from critical ischemia, 76.6% had CAD, 85.6% were hypertensive, and 34.4% had diabetes. They had a median ABI of 0.5 and slightly decreased GFR (69.7 ± 2.6 mL/kg/1.73 m^2^). Thirty-six subjects possessed a GFR of 60 mL/min/1.73 m^2^. They had HDL cholesterol and triglycerides in the normal range between 50.1 and 126 mg/dL, and total cholesterol close to the upper limit of the reference range (199.8 mg/dL). Thirty percent were affected by unilateral limb atherosclerosis, 70% possessed bilateral involvement, and only six showed solitary PAD. Thirty-three patients were affected in two arterial beds, and 51 had polyvascular disease with three-arterial-bed involvement (Table 1). Nine participants presented high carotid atherosclerosis scores, with bilateral involvement of ≥4 principal arteries.

### 3.2. Comparison of Low vs. Moderate ABI Groups

Forty-two participants had a moderately decreased ABI > 0.4, and 48 patients had low ABI ≤ 0.4. The first group was classified as the moderate PAD group, whereas the second was the severe PAD group. Only one case in the first group had a high ABI > 1.3. The groups had comparable ages and distribution of genders. GFR was significantly higher in the moderate PAD group (*p* = 0.013), while hypertension was more prevalent among the severe PAD patients (*p* = 0.032). A median ABI value of 0.59 was measured in the first group versus 0.41 in the second, and the occurrence of critical ischemia (advanced Fontaine stages III and IV) was almost exclusively limited to the severe PAD group (43 of 45). The incidence of bilateral lower limb involvement was higher in the severe than in the moderate PAD group (77% vs. 62%), but the difference did not reach significance. CAD, unilateral and bilateral carotid atherosclerosis, and polyvascular disease occurred similarly in the two groups. There were more users of angiotensin receptor antagonists among the severe PAD cases.

**Figure 1:**
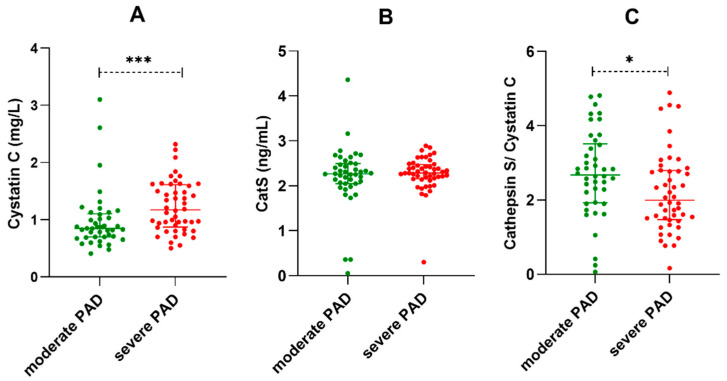
Serum Cys C (**A**), Cat S (**B**) concentrations and the Cat S/Cys C (**C**) ratio in the moderate and severe PAD groups. * *p* < 0.05, *** *p* < 0.001.

**Figure 2:**
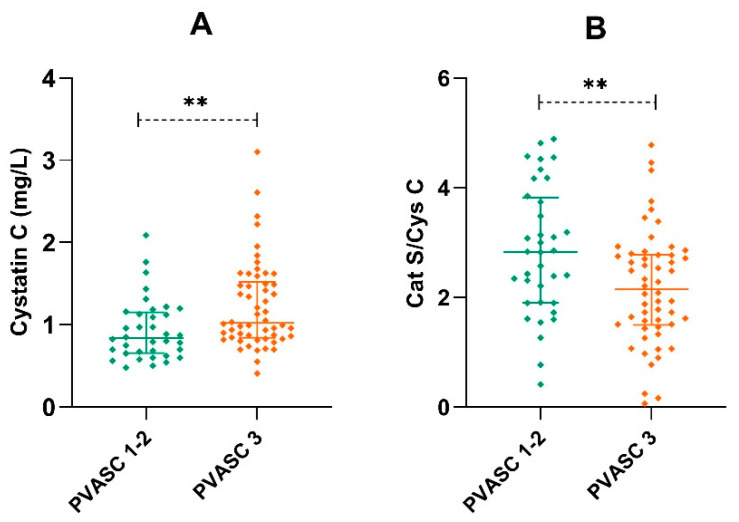
Serum Cys C concentrations (**A**) and the Cat S/Cys C ratio (**B**) in the groups with different vascular involvement. PVASC 1–2, one/two bed; PVASC 3, three bed involvement. ** *p* < 0.01.

The levels of cathepsin S were similar in the two groups. However, serum Cys C was significantly increased (Figure 1), and the CatS/CysC ratio was significantly reduced (Figure 2) in patients with low ABI (*p* < 0.001 and *p* = 0.012) (Table 1).

### 3.3. Correlations of Cys C and Cat S/Cys C

Cys C and the Cat S/Cys C ratio correlated strongly with age and GFR (*p* < 0.001 in each case). However, the ABI could be correlated only with the Cat S/Cys C ratio (*p* = 0.043). No relationship was found with the IMT for any of the variables. Instead, significant positive correlations were observed between Cys C and VWF:Ag (*p* = 0.001), Cys c and VWF:CB (*p* = 0.022). Negative associations were found between Cat S/Cys C and VWF:Ag (*p* = 0.004), Cat S/Cys C and VWF:CB (*p* = 0.031). Further, the Cat S/Cys C ratio showed significant positive correlations with the following serum lipid parameters: total cholesterol (*p* = 0.025), LDL cholesterol (*p* = 0.040) and serum triglycerides (*p* = 0.037) (Table 2).

### 3.4. Comparison of Groups with One- and Two-Bed vs. Three-Bed Atherosclerotic Involvement

The first group consisted of 39 patients with one- (six cases) or two- (33 instances) arterial-bed involvement. Fifty-one patients with three-arterial-bed involvement were enrolled in the second group. The latter were significantly older (69 vs. 63 years, *p* = 0.007), possessed a lower GFR (*p* = 0.002), and had higher HDL cholesterol (*p* = 0.035). No significant differences were seen in gender distribution or the incidence of hypertension and diabetes, and the ABI scores were only slightly but not significantly lower in the second group (0.48 vs. 0.58, *p* = 0.302). The plasma concentrations of VWF: Ag and VWF: CB showed a mild but non-significant elevation in the second group (*p* = 0.312 and *p* = 0.699). Serum Cys C was significantly higher (1.03 vs. 0.87, *p* = 0.010), and Cat S/Cys C was significantly reduced (2.08 vs. 2.80, *p* = 0.007) in the group with three-bed atherosclerosis (Table 3).

### 3.5. Predictors of Severe Peripheral Arterial Disease

Univariate regression analysis showed significant associations of hypertension (*p* = 0.029), serum Cys C (*p* = 0.001), and of the Cat S/CysC ratio (*p* = 0.006) with severe peripheral arterial disease marked by low values of the ankle–brachial index (≤0.4) (Table 4). Non-linear logistic regression models were constructed to predict low ABI in the whole study group. In the first model, when adjusted for hypertension, GFR, the presence of unilateral or bilateral PAD, and serum CRP tertiles, the odds ratio conferred by serum Cys C in the upper tertile was 6.57 (2.10–20.47), and Cys C remained a significant independent determinant of low ABI (*p* = 0.016) (Table 5, Model 1). Cys C kept its role after adjustment for angiotensin receptor antagonist usage (*p* = 0.033). The second model, designed for the Cat S/Cys C ratio, remained an important determinant of severe PAD (*p* = 0.042) after adjustment for hypertension, GFR, uni- or bilateral limb involvement (Table 5, Model 2).

### 3.6. Predictors of Polyvascular Disease with Three-Bed Involvement

First, univariate regression analysis was performed to evaluate clinical and laboratory parameters associated with polyvascular disease with three-arterial-bed involvements. Each variable was assessed, and the results showed that age (*p* = 0.042), GFR (*p* = 0.006), HDL cholesterol (0.023), serum Cys C (*p* = 0.016), and the Cat S/Cys C ratio (*p* = 0.008) were significantly correlated with the presence of three-bed atherosclerosis (Table 6). Multiple regression models were constructed for Cys C and Cat S/Cys C, where age, HDL cholesterol levels, GFR, and severity of PAD (low ABI scores) were introduced. The significant relationships disappeared with these adjustments, and neither serum Cys C nor Cat S/Cys C remained significant determinants of polyvascular disease.

## 4. Discussion

Atherosclerosis frequently manifests with multiple arterial bed involvement, which is defined now as polyvascular disease. PAD patients show a high coronary or carotid artery disease incidence, but they do not always benefit from a complex diagnostic and therapeutic management [24].

Plaque formation and development in the arteries of the lower limb, coronaries, and carotids reflect the low-grade chronic inflammation of the arterial wall and, histologically, are described as the atherosclerotic burden. An increasing number of clinical studies—Saxagliptin Assessment of Vascular Outcomes Recorded in Patients with Diabetes Mellitus (SAVOR-TIMI53), Liraglutide Effect and Action in Diabetes: Evaluation of Cardiovascular Results (LEADER), and Improved Reduction of Outcomes: Vytorin Efficacy International Trial (IMPROVE-IT)—showed that polyvascular involvement increases the risk of major adverse cardiovascular events significantly (cardiovascular death, myocardial infarction, and stroke) [15]. These studies highlighted a stepwise increase in the odds ratio for composite end-points in atherosclerotic patients with involvement of one, two, and three beds.

Due to the paucity of symptoms and the lack of proper screening tools, PAD is significantly underdiagnosed. The ankle–brachial pressure index (ABI) is an inexpensive, reliable, and relatively simple tool applicable even in primary medical care [25]. Shore et al. found that ABI and IMT were independently associated with cardiovascular disease in subjects with diabetes mellitus type 2, while pulse-wave velocity and determination of the reactive hyperemia index could not provide significant additional information [26]. More sophisticated, ultrasound-based methods were also elaborated and proposed to measure the atherosclerotic burden score (ABS) [27].

Cathepsins are essential effectors of extracellular protein remodeling with elastolytic and collagenolytic activity. In mouse models, cathepsin K, S, and L are overexpressed in MFs and SMCs of the atheromatous plaque. Cathepsin S, K, and L deficiency reduces the plaque dimensions and thickens the fibrous cap, SMC content, and collagen deposition. Some proinflammatory cytokines, IL-1β, IFN-γ, TGF-β, induce cathepsin S and L expression and stimulate elastase/collagenase activity in macrophages and smooth muscle cells [7]. Sukhova et al. compared cathepsin S−/− and LDL receptor−/− double knock-out mice with LDL receptor−/− group fed with high cholesterol diet. Double knock-out mice developed significantly decreased atheromatous plaques, less intimal thickening, and lower intimal macrophage, plaque lipid, and smooth muscle cell content. In addition, the integrity of elastic fibers in the vessel wall was higher [11]. Elastolysis and collagenolysis promote arterial inflammation and could also interfere with arterial calcification. In a mixed chronic renal disease/apo E deficiency mouse model, a high cholesterol diet produced high-level cathepsin S protein expression and increased degradation of elastin fibers. The selective cathepsin S inhibitor RO5444101 reduced these circulating markers, significantly suppressing cathepsin S and its elastolytic activity in the carotid arteries; further, cathepsin S inhibition reduced atheromatous plaque size and arterial calcification [28]. In a clinical study performed on 120 patients with high serum cathepsin S levels vs. age and sex-matched controls, the incidence of coronary artery disease and hypertension was significantly higher, and the intima-media thickness was also increased. Serum cathepsin S levels correlated strongly with the Gensini score, and patients with multi-vessel coronary artery involvement had higher Cat S than those with double-, or only single-vessel involvement [29]. Circulating forms of pro-cathepsin S, and active cathepsin S along with Cys C were higher in the plasma of abdominal aneurysm patients, and the latter two proved to be independent risk factors in multivariate logistic regression models [30]. In addition, in this study, Cat S and Cys C levels correlated negatively to ABI and positively to the aortic diameter. Cathepsin S and the Cat S/Cys C ratio was associated with cerebral infarction at day 1 and day 7 post-hospitalization in 202 stroke patients analyzed by Zhang et al. The investigators observed a decreasing trend at day 7 for Cat S and Cat S/Cys C, and, in contrast an increase at day 14, especially in lacunar cerebral infarcts [12].

Cat S was less studied in PAD than in other atherosclerotic manifestations, including its association with polyvascular disease. In recent observational research performed on type 2 diabetes subjects, Jing et al. reported higher levels of Cat S in participants with at least one atherosclerotic manifestation. However, this research did not analyze the effect of localization and polyvascular disease on Cat S values [31]. In our study, Cat S showed low variance and was similar in the groups with moderately decreased (>0.4) and low ABI (≤0.4). Patients with one- or two-bed atherosclerosis presented slightly higher Cat S values, but without a significant difference. However, the Cat S/Cys C ratios strongly correlated with age, GFR, and VWF: Ag and showed significant correlations with VWF: CB, total cholesterol, LDL cholesterol, and serum triglycerides. Cat S/Cys C was markedly decreased in cases with severe PAD (low ABI) and those with three-arterial-bed involvement. Further, the Cat S/Cys C ratio remained a significant independent predictor of severe PAD after adjustment for hypertension, GFR, and uni- or bilateral limb involvement.

In the normal aortic vessel wall, smooth muscle cells express normal quantities of Cys C. Increased aortic abdominal diameter correlates negatively with Cys C content in atherosclerotic plaque extracts [9]. Aoki et al. showed that the gene and protein expression of cathepsin B, cathepsin K, and cathepsin S is increased in aortic aneurysms, while the expression of cystatin C is decreased [32]. Moreover, in another study, smooth muscle cells and macrophages highly expressed all cysteine/aspartic proteases mentioned along with cystatin C [33].

In clinical medicine, Cys C is applied primarily as a reliable parameter of the glomerular filtration rate, but it was also shown to correlate with various measures of atherosclerotic disease. In a 5-year follow-up, Urbonaviciene et al. demonstrated that higher cystatin C levels are associated with all-cause and cardiovascular mortality among PAD patients without renal impairment [34]. In this study, higher Cys C levels were not related to critical limb ischemia or lower ABI levels, the incidence of previous myocardial infarction and cerebrovascular disease being two-fold in those with Cys C > 1 mg/L [34]. In the MESA (Multi-Ethnic Study of Atherosclerosis) study, serum Cys C, but not cystatin-based GFR, showed significant unadjusted associations with common carotid IMT (0.23 mg/L increase in Cys C was associated with 0.09 mm higher IMT). Still, this relationship was abolished after adjustment for cardiovascular risk factors [35]. However, during the 9.8-year follow-up of 6584 patients, higher cystatin baseline levels were associated with progression to low but not high ABI values in adults free of cardiovascular disease. According to the authors, the albuminuria/creatinine ratio proved to be a better predictor than serum Cys C [36]. Other results, such as those of the Cardiovascular Health Study, performed on 4025 patients, revealed the predictive role of Cys C in PAD, showing that the risk of lower extremity bypass, angioplasty, or amputation increased significantly (with a hazard ratio of 2.5) in the highest quintile (values > 1.27 mg/L) of Cys C [14]. In the study of Nakamura et al., Cys C was associated with the cardio-ankle vascular index (CAVI) in patients with cardiovascular risk factors and manifest CAD [37]. CAVI is an emerging vascular index with high plasticity, predicting future cardiovascular disease [38]; it reflects arterial stiffness and is influenced primarily by the presence of CKD and not CAD [37].

Cys C was also described as a marker of carotid atherosclerosis severity. In an observational study performed on 133 Japanese patients, the authors established the diagnostic value of serum Cys C for the maximum carotid plaque thickness of 2 mm and found an association between MCPT ≥ 2 mm with Cys C levels higher than 0.73 mg/L [39]. In our study, Cys C presented significant positive correlations with age, VWF: Ag, and VWF: CB, and a significant negative correlation with GFR. Out of 90 cases, 36 suffered from renal failure, which is frequently associated with peripheral atherosclerosis. Cys C showed a robust and significant elevation in the ABI groups in those with low scores. Univariate logistic regression analysis confirmed this correlation. Furthermore, Cys C kept its significance as a predictor of severe PAD after adjustments for hypertension, estimated GFR, unilateral- or bilateral PAD, and CRP in a logistic regression model. This result is in partial contrast with Urbonaviciene et al. [34], but these may be due to different ABI cut-off levels used for severe PAD.

Further, our results may support Garimella et al. [36], who described a relationship among high Cys C and progression to low ABI in a long-term follow-up. Cys C was also increased in subjects with three-arterial-bed involvement. However, after the adjustment of covariates, this significant relationship disappeared. Further, it is crucial that the connections mentioned above stood after adjustment and were independent of GFR.

Some studies revealed that mild chronic kidney disease associated with arterial calcification is characterized by high ABI values. Cystatin and creatinine-based GFR were decreased in persons with ABI > 1.4 compared to those with ABI between 0.9–1.1 or 1.1–1.4 [40]. Since only one subject had a high ABI in our cohort, we could not analyze this relationship.

VWF, an array of large multimeric glycoproteins [41] carries coagulation FVIII that catalyzes the coagulation cascade to form a fibrin net on the surface of the platelets, thus mediating the atherothrombotic sequelae of peripheral atherosclerosis. We observed significant, positive correlations between CysC, CatS/CysC, and VWF: Ag, VWF: CB. The VWF antigen and collagen-binding activities in this study were not increased in patients with low ABI or those with three-arterial-bed involvement. However, our previous work demonstrated that VWF: CB elevation characterizes critical ischemia and together with VWF: Ag is significantly correlated to plasma osteoprotegerin (OPG) [42]. Since OPG is a regulatory molecule related to arterial stiffness [43], VWF correlations with Cys C are significant and may characterize endothelial dysfunction.

According to the Report of the American College of Cardiology Foundation/American Heart Association Task Force on Practice Guidelines, resting ABI should be used to establish lower limb ischemia in patients with exertional leg symptoms, non-healing wounds, age 65/older, or age 50/older with history of smoking or diabetes [44]. Furthermore, measurement of ABI is recommended in both legs in PAD patients of any severity. However, ABI does not localize the anatomical segment of arterial obstruction and does not provide a reliable assessment of individuals with incompressible arteries, such as the elderly, diabetics, or patients with end-stage renal disease [44]. With a three-year follow-up of 3210 atherosclerotic subjects, the China ankle–brachial index cohort study highlighted that cardiovascular and all-cause mortality are the highest among those with ABI ≤ 0.4 [45]. For this reason, the biochemical predictors of low ABI are of particular importance. Even if Cat S concentrations alone did not provide specific information related to the stage or associations of PAD, Cys C and the Cat S/Cys C ratio may be helpful in the identification of patients with a less decreased pain-free walking distance or with no tight medical monitoring. Our study has a few limitations. First, we included a relatively reduced number of cases; therefore, further studies should confirm the results in larger cohorts. Second, our groups were not equal concerning one-bed, two-bed, and three-bed atherosclerotic involvement. For this reason, we unified the subgroups with one- and two-bed atherosclerosis and compared them to those with three-bed atherosclerosis. However, sole limb involvement would be worthwhile to characterize since it possesses the best therapeutical reserves. Third, we did not analyze the limb arteries with ultrasounds and thus could not determine the ABI scores of atherosclerotic burden. Fourth, the ABI in diabetic patients could be falsely higher than the real value because of medial calcification and arterial wall stiffness. In these cases, the Toe–Brachial index may be more relevant [44].

Our results suggest that patients with PAD should be closely monitored for the presence of carotid and coronary manifestations. In those who have a low compliance for monitoring and persistent risk factors and in subjects with poor symptomatology (silent PAD or CAD), the determination of Cys C and of the Cat S/Cys C ratio might be helpful to predict the probability of polyvascular atherosclerotic disease and low ABI. High Cys C associated with low Cat S/Cys C may warn patients and their physicians about the risk of progression to more severe forms of PAD and polyvascular disease.

## 5. Conclusions

Patients with low ABI values and polyvascular atherosclerotic manifestations have high cardiovascular mortality and need close clinical, imagistic and biochemical monitoring. In our study, Cystatin C and the Cathepsin S/Cystatin C ratio proved to be significant determinants of severe peripheral arterial disease and three-arterial-bed involvement. These markers may be helpful in the identification of potential progressors to more severe forms of atherosclerotic polyvascular involvement, even in patients with low compliance for monitoring. More studies are needed to clarify the exact pathogenetic role of CysC and Cat S in the progression of peripheral arterial disease.

## Figures and Tables

**Table 1:** Clinical variables of the 90 severe versus non-severe PAD patients.

		Group Characteristics		
Variable	All (*n* = 90)	ABI^mod^ Group (*n* = 42)	ABI^lo^ Group (*n* = 48)	*p* Value
Demographic and clinical parameters				
Age (years)	66 (57–73)	65.5 (57–72)	68.5 (58–73)	0.316
Gender (f/m)	22 (24.4)/68 (75.5)	10 (23.8)/32 (76.2)	12 (25)/36 (75)	1.000
Critical ischemia (y/n) *	45 (50)/45 (50)	2 (4.8)/40 (95.2)	43 (89.6)/5 (10.4)	<0.001
Coronary artery disease (y/n)	69 (76.6)/21 (23.4)	32 (76.2)/10 (23.8)	37 (77.1)/11 (22.9)	1.000
Hypertension (y/n)	77 (85.6)/13 (14.4)	32 (76.2)/10 (23.8)	45 (93.8)/3 (6.2)	0.032
Diabetes (y/n)	31 (34.4)/59 (65.6)	12 (28.6)/30 (71.4)	19 (39.6)/29 (60.4)	0.374
PAD of the limbs, uni- vs. bilateral (u/b)	27 (30)/63 (70)	16 (38.1)/26 (61.9)	11 (22.9)/37 (77.1)	0.166
Carotid atherosclerosis, uni- vs. bilateral (u/b)	69 (76.6)/18 (20)	32 (76.2)/7 (16.6)	37 (77.1)/11 (22.9)	0.606
Stroke history (y/n)	5 (5.5)/85 (94.5)	3 (7.1)/39 (92.9)	2 (4.1)/46 (95.9)	0.436
Polyvascular disease (y/n)	84 (93.3)/6 (6.6)	39 (92.8)/3 (7.2)	45 (93.7)/3 (6.3)	0.595
High carotid atherosclerosis score (≥4/<4)	9 (10)	3 (7.1)	6 (12.5)	0.494
Ankle–brachial index, lower	0.5 (0.39–0.73)	0.59 (0.48–0.8)	0.41 (0.32–0.56)	<0.001
Glomerular filtration rate (mL/min/1.73 m^2^) **	69.7 ± 2.6	76.9 ± 4.1	63.4 ± 3.1	0.013
Intima-media thickness (mm) ***	1.34 ± 0.10	1.29 ± 0.17	1.40 ± 0.10	0.424
Laboratory parameters				
C-reactive protein (mg/L)	3.7 (1.5–7.6)	2.7 (1.6–6.4)	4.1 (1.5–11)	0.124
Fibrinogen (mg/dL)				
Total cholesterol (mg/dL)	199.8 ± 4.4	199.8 ± 6.6	199.9 ± 6.1	0.863
Triglycerides (mg/dL)	126 (91–125)	130.2 (99–160)	121 (81–170)	0.481
HDL cholesterol (mg/dL)	50.1 ± 1.0	49.8 ± 1.5	50.7 ± 1.4	0.667
LDL cholesterol (mg/dL)	122.3 ± 3.9	123.1 ± 6.1	122.2 ± 5.0	0.945
Cystatin C (mg/L)	0.96 (0.78–1.37)	0.85 (0.70–1.09)	1.17 (0.88–1.60)	<0.001
Cathepsin S (ng/mL)	2.27 (2.12–2.48)	2.26 (2.05–2.48)	2.28 (2.16–2.46)	0.549
Cathepsin S/Cystatin C (10^−3^)	2.41 (1.61–3.08)	2.67 (1.93–3.48)	1.99 (1.48–2.78)	0.012
VWF:Ag	132 (100.8–190.1)	127.2 (100.8–178.8)	137.8 (100.6–201.5)	0.305
VWF:CB (%)	144 (105.6–178.9)	131.8 (111.7–170.8)	146.2 (96.6–192.3)	0.638
Medication				
ACE inhibitors (y/n)	49 (54.4)/41 (45.6)	24 (57.1)/18 (42.9)	25 (52.1)/23 (47.9)	0.667
Calcium blockers (y/n)	29 (32.2)/61 (67.8)	11 (26.2)/31 (75.8)	18 (37.5)/30 (62.5)	0.365
Angiotensin receptor antagonists (y/n)	18 (20)/72 (80)	3 (7.1)/39 (92.9)	15 (31.2)/33 (68.8)	0.007
Beta blockers (y/n)	25 (27.8)/65 (72.2)	13 (31.0)/29 (69.0)	12 (25.0)/36 (75.0)	0.636
Nitrates (y/n)	21 (23.3)/69 (76.7)	9 (21.4)/33 (78.6)	12 (25.0)/36 (75.0)	0.805
Antiaggregants (y/n)	84 (93.3)/6 (6.7)	40 (95.2)/2 (4.8)	44 (91.7)/4 (8.3)	0.681
Hemorheologic agents (y/n)	59 (65.6)/31 (34.4)	30 (71.4)/12 (28.6)	29 (60.4)/19 (39.6)	0.374
Statins (y/n)	66 (73.3)/24 (26.7)	29 (69.0)/13 (31.0)	37 (77.1)/11 (22.9)	0.475

Values of variables with normal distribution (marked with asterisk) are represented by the mean ± SE, whereas values of variables with abnormal distribution are shown as median (quartiles). The ABI groups (ABI^mod^ > 0.4, moderately decreased ABI; ABI^lo^ ≤ 0.4) were compared by the Mann–Whitney U test, and the Fisher’s exact test. * Critical ischemia was classified according to the Fontaine stages. ** Estimated glomerular filtration rate, calculated according to the modified MDRD formula. *** IMT was measured in 24 patients, in conformity with the methodology described in Section 2.4.

**Table 2:** Correlations of Cys C and of the Cat S/CysC ratio in the overall group.

	Cys C (*n* = 90)		Cat S/Cys C (*n* = 90)	
Variables	Spearman R	*p* Value	Spearman R	*p* Value
Age	0.555	<0.001	−0.396	<0.001
Glomerular filtration rate	−0.556	<0.001	0.526	<0.001
Ankle brachial index, lower	−0.168	0.111	0.213	0.043
Intima media thickness	−0.008	0.969	0.024	0.909
C-reactive protein	0.158	0.136	−0.169	0.110
Fibrinogen	0.021	0.841	0.022	0.835
Cathepsin S	−0.096	0.364	-	-
VWF:Ag	0.340	0.001	−0.310	0.004
VWF:CB	0.246	0.022	−0.234	0.031
Total cholesterol	−0.156	0.140	0.236	0.025
Triglycerides	−0.078	0.461	0.220	0.037
HDL cholesterol	0.054	0.613	0.014	0.894
LDL cholesterol	−0.159	0.134	0.217	0.040

Correlations were calculated with the Spearman rank correlation.

**Table 3:** Clinical variables of the 90 patients with one- or two-, versus three-bed involvement.

Group Characteristics			
Variable	PVASC^1–2^ Group (*n* = 39)	PVASC^3^ Group (*n* = 51)	*p* Value
Demographic and clinical parameters			
Age (years)	63 (54–71)	69 (62–73)	0.007
Gender (f/m)	8 (20.5)/31 (79.5)	14 (27.5)/37 (72.5)	0.267
Ankle–brachial index, lower	0.58 (0.36–0.83)	0.48 (0.39–0.61)	0.302
ABI group (low/moderate)	19 (48.7)/20 (51.3)	23 (45.1)/28 (54.9)	0.898
Critical ischemia (y/n)	19 (48.7)/20 (51.3)	26 (51)/25 (49)	1.000
Coronary artery disease (y/n)	18 (46.1)/21 (53.9)	51 (100.0)/0 (0)	<0.001
Hypertension (y/n)	31 (79.5)/8 (20.5)	46 (90.2)/5 (9.8)	0.258
Diabetes (y/n)	13 (33.3)/26 (66.6)	18 (35.3)/33 (64.7)	1.000
PAD of the limbs, uni- vs. bilateral (u/b)	18 (46.2)/21 (53.8)	9 (17.6)/42 (82.4)	0.005
Carotid atherosclerosis, uni- vs. bilateral (u/b)	34 (87.2)/5 (12.8)	35 (68.6)/16 (31.4)	0.070
Stroke history (y/n)	2 (5.4)/37 (94.6)	3 (5.9)/48 (94.1)	1.000
High carotid atherosclerosis score	2 (5.4)/37 (94.6)	7 (13.7)/44 (86.3)	0.320
Intima-media thickness (mm)	1.32 ± 0.08	1.38 ± 0.26	0.907
Glomerular filtration rate (mL/min/1.73 m^2^)	78.7 ± 4.5	62.8 ± 2.7	0.002
Laboratory parameters			
C-reactive protein (mg/L)	4.1 (1.7–6.9)	3.2 (1.3–7.8)	0.522
Fibrinogen (mg/dL)	360 (280–445)	384.5 (264.5–479)	0.971
Total cholesterol (mg/dL)	200.7 ± 7.1	199.0 ± 5.7	0.942
Triglycerides (mg/dL)	129 (96–168)	124 (82–172)	0.516
HDL cholesterol (mg/dL)	48.2 ± 1.6	51.2 ± 1.3	0.035
LDL cholesterol (mg/dL)	124.8 ± 8.9	120.9 ± 5.1	0.592
Cystatin C (mg/L)	0.87 (0.68–1.16)	1.03 (0.83–1.50)	0.010
Cathepsin S (ng/mL)	2.33 (2.15–2.56)	2.24 (2.05–2.41)	0.159
Cathepsin S/Cystatin C (×10^−3^)	2.80 (1.90–3.74)	2.08 (1.50–2.77)	0.007
VWF:Ag	128.2 (83.7–190.1)	133.9 (101.8–193)	0.312
VWF:CB (%)	139.3 (92.6–188.5)	144.7 (106.2–178)	0.699
Medication			
ACE inhibitors (y/n)	21 (53.8)/18 (46.2)	28 (54.9)/23 (45.1)	1.000
Calcium blockers (y/n)	11 (28.2)/28 (71.8)	18 (35.3)/33 (64.7)	0.498
Angiotensin receptor antagonists (y/n)	5 (12.8)/34 (95.1)	13 (25.5)/38 (74.5)	0.183
Beta blockers (y/n)	8 (20.5)/31 (79.5)	17 (33.3)/34 (66.7)	0.234
Nitrates (y/n)	11 (28.2)/28 (71.8)	10 (19.6)/41 (80.4)	0.453
Antiaggregants (y/n)	37 (94.9)/2 (5.1)	47 (92.2)/4 (7.8)	0.337
Hemorheologic agents (y/n)	32 (82.1)/7 (7.9)	27 (52.9)/24 (47.1)	0.006
Statins (y/n)	31 (79.5)/8 (20.5)	35 (68.6)/16 (31.4)	0.337

Values of variables with normal distribution (marked with asterisk) are represented by the mean ± SE, whereas values of variables with abnormal distribution are shown as median (quartiles). Continuous variables of the PAD groups were compared with the Mann–Whitney U test, whereas the categorical variables with the Fisher’s exact test. PVASC^1–2^, one or two arterial beds; PVASC^3^, three-arterial-bed involvement.

**Table 4:** Univariate logistic regression of variables associated with low ABI (≤0.4).

Variables	Coefficient	SD	*p* Value	OR (95% CI)
Hypertension	1.554	0.697	0.029	4.68 (1.19–18.40)
Glomerular filtration rate	0.719	0.441	0.107	2.05 (0.86–4.87)
Peripheral arterial disease, unilateral/ bilateral	0.727	0.468	0.123	2.07 (0.82–5.17)
CRP, tertile 3: tertile 1	0.269	0.261	0.304	1.72 (0.61–4.84)
Angiotensin receptor antagonist usage (y/n)	1.750	0.675	0.011	5.13 (1.37–19.16)
Cystatin C, tertile 3: tertile 1	0.933	0.287	0.001	6.57 (2.10–20.47)
Cathepsin S/Cystatin C	−0.773	0.278	0.006	0.21 (0.07–0.63)

**Table 5:** Summary of multiple logistic regression analysis of the factors correlated to severe PAD (ABI ≤ 0.4) in the overall patient group (*n* = 90).

Model 1		
Variables	Odds Ratio (95% CI)	*p* Value
Cystatin C (mg/L), tertile 3: tertile 1	6.57 (2.10–20.47)	0.016
Hypertension (yes/no)	4.68 (1.19–18.40)	0.064
Glomerular filtration rate (<60 mL/min/1.73 m^2^ ≥60 mL/min/1.73 m^2^)	2.05 (0.87–4.87)	0.765
Peripheral arterial disease, bilateral: unilateral	2.07 (0.82–5.17)	0.203
C-reactive protein (mg/L) tertile 3: tertile 1	1.72 (0.61–4.84)	0.251
**Model 2**		
**Variables**	**Odds Ratio (95% CI)**	***p* Value**
Cathepsin S/Cystatin C, tertile 3: tertile 1	0.21 (0.07–0.63)	0.042
Hypertension (yes/no)	4.68 (1.19–18.40)	0.065
Glomerular filtration rate (<60 mL/min/1.73 m^2^ ≥60 mL/min/1.73 m^2^)	2.05 (0.87–4.87)	0.650
Peripheral arterial disease, bilateral: unilateral	2.07 (0.82–5.17)	0.341

**Table 6:** Summary of univariate logistic regression of variables associated with polyvascular disease (*n* = 3 arterial beds involved).

Variables	Coefficient	SD	*p* Value	OR (95% CI)
Age	0.557	0.270	0.042	2.78 (1.12–6.87)
Glomerular filtration rate	1.321	0.472	0.006	3.75 (1.48–9.46)
HDL cholesterol	0.631	0.273	0.023	2.78 (1.12–6.87)
Hemorheologic agent usage (y/n)	−1.462	0.502	0.006	0.246 (0.091–0.659)
Cystatin C	1.352	0.553	0.016	2.10 (0.89–4.95)
Cathepsin S/Cystatin C	−0.564	0.210	0.008	3.73 (1.39–10.09)

## Data Availability

Data spreadsheets are available as Nagy, Előd Ernő (2021), “Cathepsin S- Cystatin C data- PAD-polyvascular disease”, Mendeley Data, V1, doi:10.17632/zjw625jj3w.1.

## Abbreviations

ABI	ankle–brachial index
ACE	angiotensin convertase
Apo E	apolipoprotein E
CAD	coronary artery disease
CAPRIE	clopidogrel versus aspirin in patients at risk of ischemic events
CAT S	cathepsin S
CAVI	cardio-ankle vascular index
CRP	C-reactive protein
CRUSADE	Can Rapid Risk Stratification of Unstable Angina Patients Suppress Adverse Outcomes with Early Implementation of the ACC/AHA Guidelines
CYS C	cystatin C
ECST	European Carotid Surgery Trial
ELISA	enzyme-linked immunosorbent assay
EUCLID	Examining Use of Ticagrelor in Peripheral Artery Disease
GFR	glomerular filtration rate
HDL	high density lipoprotein
HsCRP	high sensitivity C-reactive protein
IFN-γ	interferon γ
IL-1β	interleukin-1 β
IL-6	interleukin-6
IMT	intima-media thickness
JNC	Joint National Committee on Prevention, Detection, Evaluation, and Treatment of High Blood Pressure
LDL	low-density lipoprotein
MCPT	maximum carotid plaque thickness
MDRD	Modification of Diet in Renal Disease
MF	macrophage
NLRP3	NOD-, LRR- and pyrin domain-containing protein 3
OPG	osteoprotegerin
PAD	peripheral arterial disease
PEGASUS-TIMI54	Prevention of Cardiovascular Events in Patients with Prior Heart Attack Using Ticagrelor Compared to Placebo on a Background of Aspirin–Thrombolysis in Myocardial Infarction 54
PVASC	polyvascular disease
REACH	Research to Assess SVS Spiration^®^ Valve System
SMC	smooth muscle cells
TGF-β	transforming growth factor β
VWF	Von Willebrand Factor Antigen
VWF	Von Willebrand Factor collagen-binding activity
